# α_2_-adrenoceptor-mediated inhibition in the central amygdala blocks fear-conditioning

**DOI:** 10.1038/s41598-017-12115-x

**Published:** 2017-09-15

**Authors:** N. M. Holmes, J. W. Crane, M. Tang, J. Fam, R. F. Westbrook, A. J. Delaney

**Affiliations:** 1 School of Biomedical Sciences, Charles Sturt University, Orange, NSW 2800 Australia; 2 School of Psychology, University of New South Wales, Sydney, NSW 2052 Australia

## Abstract

The central amygdala is critical for the acquisition and expression of fear memories. This region receives a dense innervation from brainstem noradrenergic cell groups and has a high level of α_2_-adrenoceptor expression. Using whole-cell electrophysiological recordings from rat brain slices, we characterise the role of pre-synaptic α_2_-adrenoceptor in modulating discrete inhibitory and excitatory connections within both the lateral and medial division of the central amygdala. The selective α_2_-adrenoceptor agonist clonidine blocked the excitatory input from the pontine parabrachial neurons onto neurons of the lateral central amygdala. In addition, clonidine blocked inhibitory connections from the medial paracapsular intercalated cell mass onto both lateral and medial central amygdala neurons. To examine the behavioural consequence of α_2_-adrenoceptor-mediated inhibition of these inputs, we infused clonidine into the central amygdala prior to contextual fear-conditioning. In contrast to vehicle-infused rats, clonidine-infused animals displayed reduced levels of freezing 24 hours after training, despite showing no difference in freezing during the training session. These results reveal a role for α_2_-adrenoceptors within the central amygdala in the modulation of synaptic transmission and the formation of fear-memories. In addition, they provide further evidence for a role of the central amygdala in fear-memory formation.

## Introduction

The central amygdala (CeA) receives processed and unprocessed sensory input carrying information about both the conditioned stimulus and unconditioned stimulus needed for fear conditioning. It is also extensively connected (via inhibitory projections) to the brainstem and hypothalamic nuclei responsible for the behavioural, hormonal and autonomic changes that comprise the conditioned response^1^. Recent studies have shown that the excitatory connection between the pontine lateral parabrachial nucleus (PBr) and the lateral division of the central amygdala (CeAL) is involved in the acquisition of context conditioning in mice^2^, potentially conveying information related to the unconditioned stimulus needed for fear-conditioning to take place. Our previous research characterised this connection and found that it provided supra-threshold excitation of the CeAL cells and that this input was regulated by the local release of noradrenaline, activating presynaptic α_2_-adrenoceptors on the PBr basket terminals^3^.

Immunohistochemical and tract tracing studies, have provided a comprehensive picture of noradrenergic innervation of the CeA. Noradrenergic projections arise primarily from the A6 cell group of the locus coeruleus, distributing noradrenergic terminals throughout the amygdala. The medial division of the central amygdala (CeAM) receives particularly dense noradrenergic projections^4^ and we have previously demonstrated noradrenergic terminal labelling in close proximity to PBr terminals within the CeAL^3^. Consistent with this dense innervation, noradrenaline is released into the CeA in rats in response to stressors^5,6^. The CeA has been shown to express both α-adrenoceptors: α_1_A and α_2_A mRNA has been detected in both the medial and lateral divisions^7,8^, and immunolabelling has revealed α_2_A and α_2_C adrenoceptors are present in CeAL^9,10^.

Despite the anatomical evidence for noradrenergic projections into the CeA, the role of noradrenaline in modulating neuronal activity within the CeA has not been widely investigated. There are reports that activation of noradrenergic receptors in CeA produces hypoalgesia^11,12^, increases drug-seeking behaviour^13^ and change anxiety-like behaviours following acute stress^14^. However, little is known about how noradrenaline modulates the circuit activity in the CeA, or how this might play a role in fear-conditioning. In this study, we have examined how α_2_-adrenoceptor activation alters excitatory and inhibitory synaptic activity within the CeAM and CeAL, and whether activation of α_2_-adrenoceptor within the CeA influences the acquisition of fear-conditioning in rats.

## Results

We first tested whether the selective α_2_-adrenoceptor agonist clonidine had direct effects on the various cell types in the CeAL and CeAM. Whole-cell electrophysiology recordings were made using a potassium-based internal solution (see methods) in the presence of both NBQX and picrotoxin to block both excitatory and inhibitory synaptic transmission, respectively. Based on the firing properties of the neurons, we confirmed previous reports^15^ of two types of CeAL cells (repetitive-firing neurons and adapting neurons) and two types of neurons in the CeAM (single-firing neurons and adapting neurons). Clonidine administration had no effect on the input resistance, action potential threshold or action potential half-width displayed by the two cells types in the CeAL or the adapting neurons of the CeAM (Table 1). Clonidine administration produced a significant decrease in input resistance of single-firing neurons of the CeAM, but both action potential threshold and half-width were unaffected (Table 1).

**Table 1:** The effect of 10 µM clonidine on membrane and firing properties of CeAL and CeAM neurons.

sub-division	Cell type	number of cells	resting membrane potential (mV)	input resistance (MΩ)		Action potential threshold (mV)		Action potential width (ms)	
				*ctl*	*clon*	*ctl*	*clon*	*ctl*	*clon*
CeAL	Adapting	9	−64.9 ± 4.1	395 ± 208	308 ± 161	−32.6 ± 3.8	−32.7 ± 6.3	0.96 ± 0.14	0.92 ± 0.14
	Repetitive	11	−64.9 ± 5.3	369 ± 152	431 ± 174	−38.0 ± 3.4	−38.2 ± 2.8	1.02 ± 0.22	0.96 ± 0.15
CeAM	Single	8	−55.7 ± 6.5	520 ± 85	376 ± 296*	−35.4 ± 8.1	−36.4 ± 7.9	1.00 ± 0.23	1.06 ± 0.21
	Adapting	8	−60.9 ± 4.1	291 ± 85	263 ± 81	−33.4 ± 10	−35.3 ± 11.4	0.82 ± 0.12	0.87 ± 0.17
				*p < 0.05					

**Figure 1:**
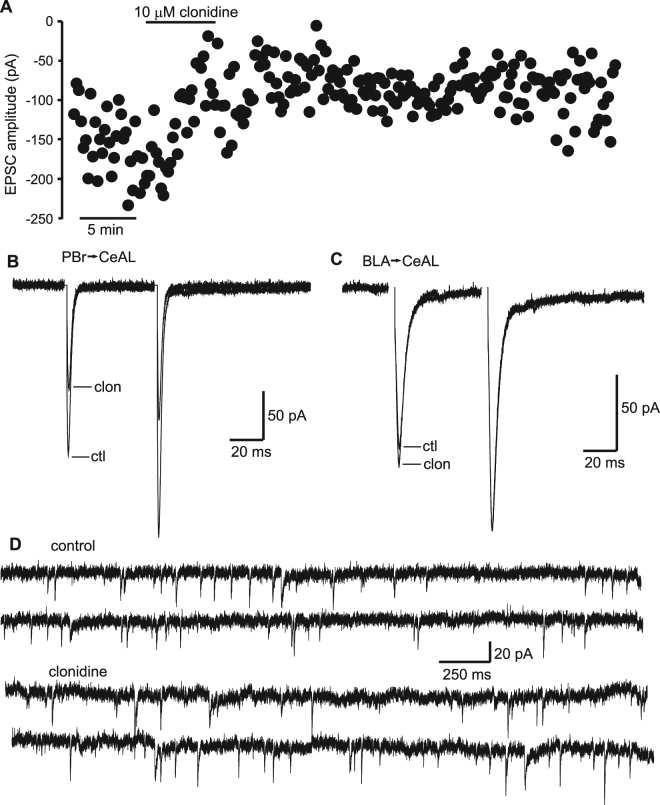
Parabrachial-evoked EPSCs but not BLA-evoked EPSCs are blocked by clonidine in the CeAL. (**A**) Scatterplot of EPSC amplitudes recorded from a CeAL neuron in response to stimulation of the PBr input, with addition of clonidine to the perfusate shown (Bar) and a long washout following. (**B**) Representative PBr-evoked EPSC recorded from the CeAL neuron shown in A, in control (ctl) and after application of 10 µM clonidine (clon). (**B**) Representative BLA-evoked EPSC in control (ctl) and after application of 10 µM clonidine (clon). Averages in (**B**) and (**C**) are from 24 individual responses in each condition. (**D**) Representative traces showing spontaneous EPSCs recorded from a CeAL cell in control conditions and after application of clonidine.

We next sought to characterise the effect of clonidine on synaptic transmission within the CeAL. Confirming our previous results with noradrenaline^3^ we found that excitatory post-synaptic currents (EPSCs) recorded in CeAL cells in response to stimulation of the PBr axons medially to the CeAL were inhibited by clonidine (Fig. 1A,B), with 43.1 ± 2.7% inhibition (p < 0.05, n = 12) in 10 µM clonidine and 75.8 ± 2.8% inhibition in 20 µM clonidine (p < 0.05, n = 5). Furthermore, clonidine-mediated inhibition of the EPSC response to PBr-terminal stimulation did not involve a change in the paired pulse ratio (PPR) of this input (4.6 ± 8.1% change at 20 µM (p > 0.05, n = 5). The effect of clonidine on release at the PBr synapses persisted for more than 45 min during washout (Fig. 1A), and only partial recovery was observed. In contrast, EPSCs recorded in CeAL cells in response basolateral amygdala (BLA) complex stimulation in the lateral amygdala (LA) were not affected by clonidine (0.0 ± 9.6% change at 10 µM (Fig. 1C; p > 0.05, n = 6). Despite the inhibition of the PBr-CeAL EPSCs, clonidine did not affect amplitude or frequency of spontaneous EPSC (sEPSC) recorded from CeAL cells (Fig. 1C; 0.9 ± 6.9% change in frequency, 1.1 ± 4.2% change in amplitude (p.0.05 for both, n = 12). This result is consistent with anatomical distribution of these excitatory inputs. The PBr input makes a single large connection onto the soma of CeAL cells^16^. Therefore, it is likely that any change in frequency or amplitude of spontaneous release events from this single PBr-CeAL connection was undetectable within the background of spontaneous release from unaffected excitatory connections to the CeAL neurons.

**Figure 2:**
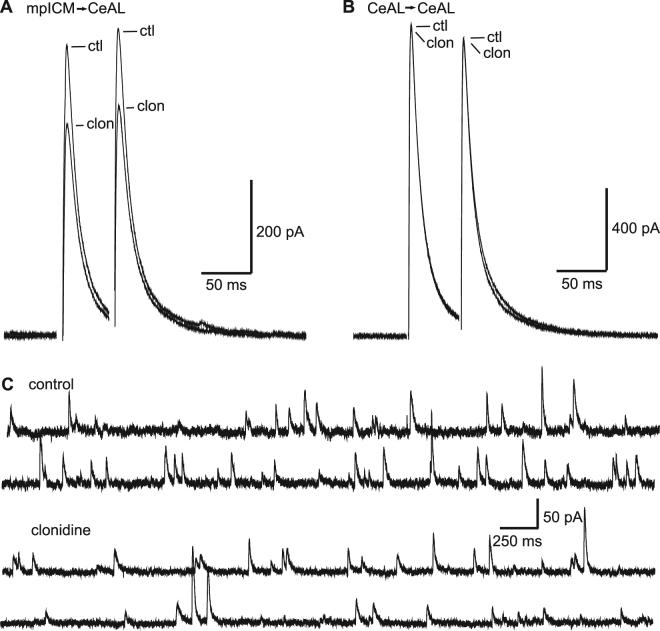
mpICM-evoked IPSCs but not local CeAL-evoked IPSCs are blocked by clonidine in the CeAL. (**A**) Representative mpICM-evoked IPSC recorded from a CeAL neuron in control (ctl) and after application of 10 µM clonidine (clon). (**B**) Representative IPSCs stimulated locally within the CeAL in control (ctl) and after application of 10 µM clonidine (clon). Averages in (**A**) and (**B**) are from 24 individual responses in each condition. (**C**) Representative traces showing spontaneous IPSCs recorded from a CeAL cell in control conditions and after application of clonidine.

Next, we characterised the effects of clonidine (10 µM) on inhibitory transmission in the CeAL. Electrically-evoked monosynaptic IPSC responses were recorded from CeAL cells at depolarised membrane potentials (−10 to 0 mV) in the presence of 10 µM NBQX to block glutamatergic transmission. We first stimulated electrically at the internal capsule boundary of the LA to activate connections from medial paracapsular intercalated cells (mpICM) from the anterior intercalated island^17^. Clonidine inhibited IPSCs evoked by mpICM stimulation (Fig. 2A; 35.3 ± 4.7% inhibition, n = 7, p < 0.05) without changing PPR (2.92 ± 6.7%, n = 7, p > 0.05). In contrast, IPSCs recorded from CeAL neurons in response to local stimulation within the CeAL (most likely connections between CeAL cells) were not affected by clonidine (Fig. 2B; 1.8 ± 5.0% change, n = 5, p > 0.05). Clonidine also significantly reduced the frequency (14.3 ± 4.8% p < 0.01, n = 11) and amplitude (9.2 ± 4.3%, p < 0.05, n = 11) of spontaneous IPSCs recorded from CeAL neurons (Fig. 2C). Together, these results suggest that a significant proportion of mpICM-derived inhibitory-inputs (but not connection from local inhibitory-neurons) onto CeAL neurons are inhibited by clonidine. Furthermore, these results suggest that a significant level of basal inhibitory activity in the CeAL results from mpICM-input into the CeAL.

**Figure 3:**
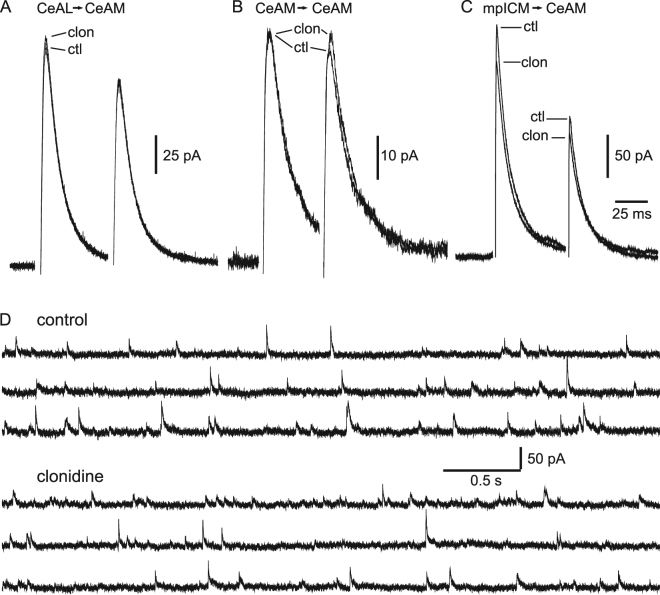
mpICM-evoked IPSCs but not local CeAM-evoked IPSCs or CeAL-evoked IPSCs are blocked by clonidine. Representative IPSCs stimulated in the CeAL (**A**) or locally within the CeAM (**B**) in control (ctl) and after application of 10 µM clonidine (clon). (**C**) Representative ICM-evoked IPSC recorded from a CeAM neuron in control (ctl) and after application of 10 µM clonidine (clon). Averages in (**A,B** and **C**) are from 24 individual responses in each condition. (**C**) Representative traces showing spontaneous IPSCs recorded from a CeAM cell in control conditions and after application of clonidine.

Finally, we characterised the effects clonidine on evoked EPSCs and IPSCs recorded from CeAM neurons. Clonidine had no significant effect on excitatory currents evoked by stimulating in the BLA (representative data not shown; 0.2 ± 10.9% change, p > 0.05, n = 5). The frequency and amplitude of spontaneous EPSCs were also unaffected by clonidine (0.5 ± 5.5% and 5.2 ± 7.6% change respectively, p > 0.05, n = 8, representative data not shown). In addition, clonidine had no effect on inhibitory responses recorded from CeAM neurons in response to stimulation of CeAL neurons (Fig. 3A; 2.2 ± 6.4% change, p > 0.05, n = 7) or in response to local stimulation in the CeAM (0.8 ± 5.2% change, (Fig. 3B; p > 0.05, n = 4). In contrast, clonidine did reduce the amplitude of IPSCs recorded from CeAM neurons in response to stimulation of the internal capsule (Fig. 3C; 9.7 ± 7.5% inhibition, p < 0.05, n = 6). These responses most likely result from activation of inhibitory input from ventrally-located mpICM^18^. In contrast to CeAL neurons, clonidine did not change the frequency or amplitude of spontaneous IPSCs recorded from CeAM (Fig. 3D; 8.0 ± 8.9% and 1.4 ± 7.6% change respectively, p > 0.05, n = 16).

**Figure 4:**
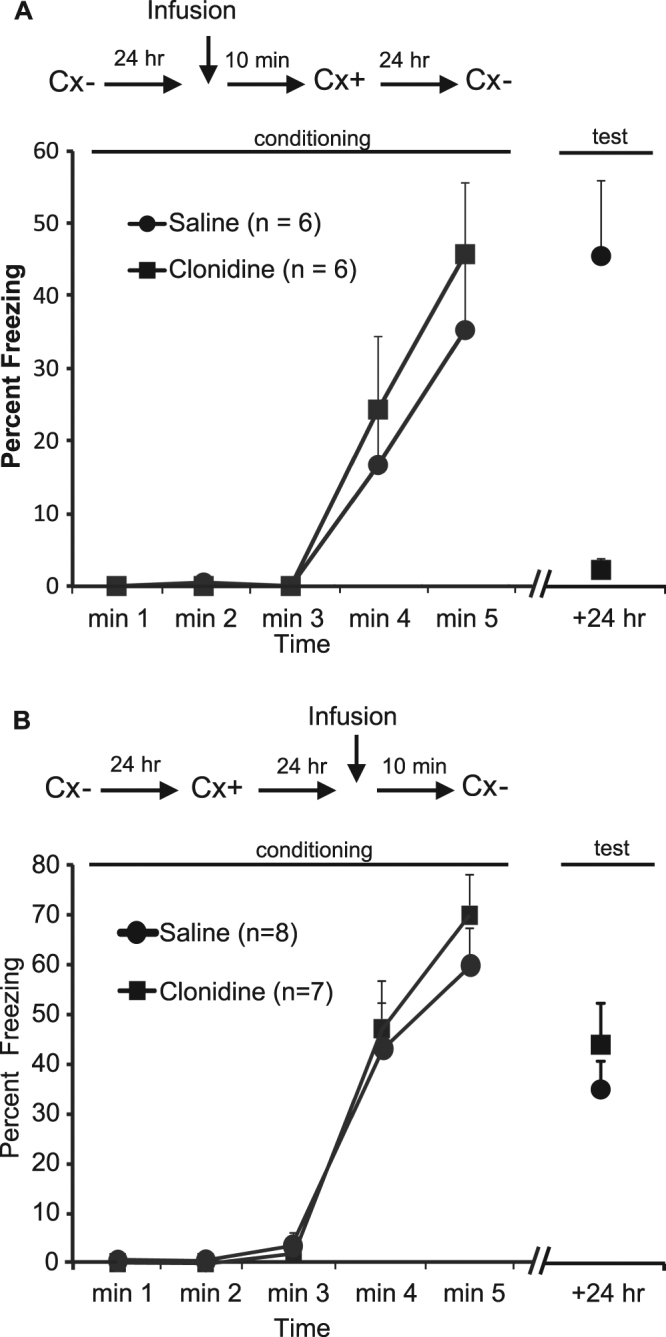
Clonidine blocks the acquisition of contextual fear. (**A**) Schematic outlines the experimental design with clonidine infusions occurring 10 mins prior to the onset of training in the context co-inciding with footshock shock and freezing. The graph shows the mean ± s.e.m. level of freezing in 1 min intervals during the aquisition session and in a 2 min test-period 24 hours later for Groups Clonidine and Saline. (**B**) shows the experimental design with clonidine infusions occurring 10 mins prior to the onset of testing in the context 24 hours after acquisition. The graph shows the mean ± s.e.m. level of freezing in 1 min intervals during the acquisition session and in a 2 min test-period 24 hours later for Groups Clonidine and Saline.

These results demonstrate that the effects of clonidine within the CeA are quite discrete, affecting only the excitatory input to the CeAL from the PBr, and the inhibitory input from the medial paracapsular intercalated cells. To assess their functional significance, we examined the effect of CeA clonidine infusions on different aspects of context conditioned fear in rats. We first examined the effect of these infusions on acquisition of the context fear memory. In this experiment, rats received a CeA infusion of clonidine or vehicle immediately prior to pairings of a distinctive context and foot shock. One day later, they were tested for their levels of freezing (an index of conditioned fear) in the context. The infusion of clonidine into the CeA had no effect on the acquisition of freezing responses to the context, but impaired the retention of these responses over time (Fig. 4A). Across conditioning, there was a significant linear increase in freezing across the session (*F*_(1,10)_ = 22.79, *p* = 0.001, η_p_^2^ = 0.70), but the rate at which freezing increased and the overall levels of freezing did not significantly differ between the two groups (*F*_s_ < 1). In contrast, in the drug-free test session that occurred 24 hours later, the levels of freezing were significantly lower in clonidine-infused animals compared to saline-infused animals (*F*_(1,10)_ = 22.52, *p* = 0.001, η_p_^2^ = 0.69).

The second experiment examined the effects of CeA clonidine infusions on expression of context conditioned fear. Here, rats were again exposed to pairings of a distinctive context and foot shock. The next day, they received a CeA infusion of clonidine or vehicle before being returned to the context for a brief session of context alone exposure. The infusion of clonidine into the CeA had no effect on the expression of freezing responses to the context (Fig. 4B). Across conditioning, there was a significant linear increase in freezing across the session (*F*_(1,13)_ = 120.08, *p* < 0.001, η_p_^2^ = 0.90), but the rate at which freezing increased and the overall levels of freezing did not differ between the two groups (*F*_s_ < 1). When animals were tested immediately after clonidine or vehicle infusion, there was no significant difference in freezing between the two groups in the first 2 minutes. (*F*_s_ < 1).

## Discussion

In this study, we have characterised the synaptic connections received by CeA neurons that are modulated by the activation of α_2_-adrenoceptors. These connections are the ascending excitatory input from the PBr, activating neurons in the CeAL, and inhibitory connections arising the mpICMs^19^ onto both CeAL and CeAM neurons. The effect of clonidine infusions into the CeA was to prevent contextual fear conditioning, though increased freezing during the training session indicated that an association between the context and footshock was being made during this session. This implies that inputs from the PBr or the mpICMs, or both, are required for the consolidation of contextual fear memories.

The PBr-CeA pathway has been shown to be crucial for delivering unconditioned stimulus information during fear conditioning. Inactivation of the lateral PBr by muscimol^20^ or genetic silencing of the CeAL projecting PBr^2^ cells blocks the acquisition of fear memory in mice. Further, direct, optogenetic activation of the PBr-CeAL input can induce fear learning when paired with a conditioned stimulus^20^. Han *et al*.^2^ reported that genetic silencing of both release from PBr terminals in the CeA and neurons within the CeA attenuated the acquisition of contextual fear. This differs from our results where *in vivo* infusion of clonidine into the CeA that would, in part, have inhibited release from PBr terminals had no effect on the acquisition of contextual fear but inhibited consolidation of this memory. The difference between our results and the results of Han *et al*. is most likely due to differences in the level of neuronal inhibition produced. The genetic silencing used by Han *et al*. produced a complete block of the input from PBr onto CeA neurons. However, our *in vitro* results have repeatedly demonstrated that maximum α2-mediated-inhibition of the PBr input onto CeAL neurons is around 75%^3^. Therefore, we conclude that the partial inhibition of this input by clonidine allows sufficient information about the unconditioned stimulus to enter the CeA required for acquisition, but provides enough block of unconditioned-stimulus information during conditioning to interfere with the consolidation of the contextual fear memory. Alternatively, the fact that the acquisition-phase was unaffected by clonidine could result from alternate pathways for unconditioned-stimulus input into the amygdala, such as thalamic pathways to the BLA^20^, that are sufficient to evoke freezing behaviours and acquisition of contextual fear.

There have been several studies indicating that the mpICMs play a role in the fear extinction process^21–23^, and others indicating that sub-populations of ICMs may also participate in fear expression^24,25^. Recent studies have also suggested that ICM neurons may also play a role in fear memory acquisition^25,26^. In the present study, we have demonstrated that clonidine has an inhibitory effect on inhibitory input from the mpICMs to both the neurons of the CeAM and CeAL, and blocks consolidation of fear memory when delivered into the CeA. These results suggest that α_2_-adrenoceptor-mediated regulation of mpICM inputs to the CeA may play an important role in modulating the formation of fear memories.

In the present study, a pre-training infusion of clonidine into the CeA had no acute effect on the acquisition of a context fear memory, but disrupted its retention over time. One possible explanation for this result is that a pre-training clonidine infusion establishes a unique ‘state’ that competes with the context for association with the aversive foot shock (i.e., it overshadowed the context): hence, the absence of this ‘state’ at test (which was conducted in the absence of clonidine) results in lower levels of freezing in the context. However, if this was the case, pre-testing infusions of clonidine might have been expected to generate the same behavioural effect: that is, we would expect a similar lack of freezing among rats trained drug-free and then tested under the influence of clonidine. Contrary to this prediction, clonidine spared the expression of an already-formed context fear memory: rats trained drug-free but tested immediately after clonidine-infusions froze to the same extent as saline-infused animals. This leads us to conclude that the effect of clonidine on a context fear memory is not due to state-dependent effects of the drug. Instead, the overall pattern of results suggests that CeA clonidine infusions affect the stabilization, or *consolidation*, of a context fear memory. This is consistent with our *in vitro* results demonstrating that the effects of clonidine persist for up to 45 min after drug application has stopped. It is sometimes argued that the effects of a given treatment on the consolidation of a memory (fear or other) can be distinguished from its effects on acquisition by administering the treatment immediately after the training session. While post-training administration of drugs into discrete regions of the amygdala has been shown to disrupt retention of recently formed fear memories, other studies have reported patterns of results that are very similar to those obtained in the present study. For example, pre-training inhibition of protein kinase signalling pathways in the amygdala has been shown to spare the acquisition but disrupt the consolidation of fear memories^27,28^. The simplest explanation of these findings, as well as our own, is that processes required for consolidation of a fear memory are initiated at the time of its acquisition (i.e., at the time of context-shock pairings), but are distinct from those required for that acquisition. This explanation is consistent with the findings of the present study where clonidine-infusion into the CeA did not influence the formation of the fear memory but selectively inhibited its consolidation.

Finally, the present results extend recent reports that systemic administration of clonidine interferes with consolidation and reconsolidation of conditioned fear memories^29,30^. These effects of clonidine have been attributed to an α_2_-adrenoceptor-mediated inhibition of noradrenaline release from terminals of locus coeruleus noradrenergic neurons, leading to a reduction of β-adrenoceptor activation in the BLA^30^. Consistent with this proposal, systemic administration of clonidine completely suppresses foot shock-induced release of noradrenaline in the amygdala^31^, and injections of clonidine directly into the BLA blocks both the acquisition and expression of fear-potentiated startle^32^. Our findings suggest that the effects of systemically administered clonidine might also reflect mechanisms of action in the CeA, including α_2_-adrenoceptor-mediated inhibition of PBr and mpITC inputs to CeAL and CeAM neurons (see also refs^2,24,25^).

## Methods

### Electrophysiology

#### Acute Slice preparation

Coronal brain sections were prepared from 28–35 day old Sprague-Dawley rats supplied by Animal Resources Centre, Perth, Western Australia. Rats were anaesthetized using isoflurane, decapitated, and their brains removed into ice cold artificial cerebrospinal fluid (ACSF) solution containing (in mM): 118 NaCl, 25 NaHCO_3_, 10 Glucose, 0.5 CaCl_2_, 1.2 NaH_2_PO_4_ and 2.6 MgCl_2_. Brains were sliced into 350 µm thick coronal sections using a Leica VT1000S vibratome at 0 °C, transferred to a chamber containing ACSF at 33–34 °C for 30 mins, then maintained for several hours in ACSF at room temperature. All experimental procedures were approved by the Animal Care and Ethics Committee at the Charles Sturt University and performed in accordance with the National Institute of Health *Guidelines for the Care and Use of Laboratory Animals*, revised 1996.

#### Whole-cell recording

Whole-cell recordings were made from brain slices maintained at 32–33 °C in a recording chamber continuously perfused with oxygenated ACSF solution containing (in mM): 118 NaCl, 25 NaHCO_3_, 10 Glucose, 2.5 CaCl_2_, 1.2 NaH_2_PO_4_ and 1.3 MgCl_2_, and visualized using IR/DIC techniques. In voltage-clamp recordings, patch electrodes (3–5 MΩ) were filled with pipette solution containing (mM): CsMeSO_4_ 135, NaCl 8, HEPES 10, Mg_2_ATP 2 and Na_3_GTP 0.3 (pH 7.2 with CsOH, osmolarity 290 mOsm/kg). KMeSO_4_-based pipette solution containing (in mM); KMeSO_4_ 135, NaCl 8, HEPES 10, Mg_2_ATP 2 and Na_3_GTP 0.3 (pH 7.2 with KOH, osmolarity 285 mOsm/kg) was used for current-clamp recordings.

Whole-cell recordings were made using a patch-clamp amplifier (Multiclamp 700B, Axon instruments, Foster City, CA). Current and voltage signals were filtered at 4–8 kHz and digitized at 20 kHz (National Instruments, USB-6221 digitiser), acquired, stored and analyzed on Toshiba Satellite Pro L70 PC using Axograph software. Access resistance (5–15 MΩ) was monitored throughout the experiment.

Drugs were added to the ACSF solution used to perfuse the slice, including picrotoxin, NBQX, and clonidine (Abcam). Evoked post-synaptic currents were stimulated using a bipolar electrode rotated perpendicular to the slice with a single electrode tip placed onto the surface of the slice. Responses shown are averages of 10–50 individual trials. Student’s t*-*tests were used for statistical comparisons between groups. All results are expressed as mean ± s.e.m.

### Contextual Fear conditioning

#### Subjects

Subjects were 32 experimentally naïve, male, Sprague-Dawley rats (weighting between 320 g to 400 g). Rats were obtained from a commercial supplier (Animal Resources Centre, Perth, Western Australia). The subjects were housed in plastic boxes (67 cm length × 40 cm width × 22 cm height) in groups of four. Rats had free access to food and water. Housing boxes were located in a climate controlled colony room maintained on a 12:12 light/dark cycle (lights on from 7:00 A.M. to 7:00 P.M.). Each rat was handled for approximately three minutes each day for seven days prior to the commencement of an experiment. All experimental procedures were approved by the Animal Care and Ethics Committee at the University of New South Wales and in accordance with the National Institute of Health *Guidelines for the Care and Use of Laboratory Animals*, revised 1996. The 16 animals were randomly and equally assigned to one of two treatment conditions: Drug (Clonidine), or Vehicle (Saline).

#### Behavioural Apparatus

All behavioural training and testing was conducted in a set of four chambers (30 cm length × 27 cm width × 30 cm height). The front and back wall of each chamber was made of clear Perspex, and the sidewalls and ceiling were made of aluminium. The floor of each chamber consisted of stainless steel rods, 2 mm in diameter and spaced 13 mm apart (centre to centre). A tray below the floor contained corncob bedding material. Each chamber was enclosed in a sound- and light-attenuating cabinet whose floor, ceiling, and sidewalls were painted black. An infrared light source (940 ± 25 nm) and a camera (Sony CCD 420TVL) were mounted on the back wall of each cabinet. The latter was used to record the behaviour of each rat during training and test sessions. The cameras were connected to a monitor and DVD recorder located in an adjacent room. This room also contained a computer with appropriate software (MATLAB, MathWorks Inc.) for programming experimental events (i.e., foot-shock), and an interface for relaying the necessary signals to each conditioning chamber. The background noise level of the room was 50 dB measured by a digital sound-level meter (Dick Smith Electronics, Australia).

A custom-build constant-current shock generator, capable of delivering unscrambled alternating current (AC) 50 Hz shock to the floor of each chamber, was used for the presentation of a 0.5 s duration shock at 0.8 mA intensity. All chambers were cleaned using water at the end of each training and test session.

#### Surgery

Rats were anaesthetized with 100 mg/ml of ketamine (Ketapex; Apex Laboratories, Sydney, Australia), administered at a dose of 1.0 ml/kg (i.p.), mixed with dosage of 0.3 ml/kg of the muscle relaxant, xylazine (Rompun; Bayer, Sydney, Australia) at a concentration of 20 mg/ml. The anesthetized animals were placed in a stereotaxic apparatus (Kopf Instruments, Tujunga, CA), and received intracranial surgery. Guide cannulas (26 gauge, 11 mm in length, Plastics One, Roanoke, VA) were implanted bilaterally targeting the CeA of both hemispheres according to the following coordinates from the rat brain atlas^33^: 2.3 mm posterior to bregma, 4.2 mm lateral to the midline, and 8.0 mm ventral to the skull. The guide cannulas were secured to the skull with dental cement and four jeweller’s screws. A dummy cannula was kept in each guide at all times. These extended approximately 1 mm beyond the end of the guide cannula. They were removed for microinjections and replaced immediately afterwards. Following surgery, all animals received an i.p. injection of a prophylactic (0.3 ml) dose of 300 mg/kg solution of procaine penicillin. The rats were allowed seven days to recover from surgery, prior to experimentation. During this time, all animals were handled two to three minutes each day, and monitored for any weight and/or behavioural changes.

#### Drug Infusions

The α_2_-adrenoreceptor agonist clonidine (Sigma, Australia) was dissolved in non-pyrogenic saline (concentration) to a final concentration of 6.25 μg/μL^34^. A total volume of 0.2 μL of this solution was infused into the CeA in each hemisphere via a 33-gauge internal infusion cannula that was inserted into each guide cannula. The infusions lasted one minute and the infusion cannulas remained in place for two minutes post-infusion to allow for diffusion of the drug or vehicle. The interval between the removal of the infusion cannulae and the placement of rats in the experimental chamber for training or testing was exactly seven minutes.

#### Procedure

*Pre-exposure*. On Day 1, rats were exposed to the experimental chambers for five minutes in order to reduce any neophobia and to increase context conditioned fear^35,36^.

*Acquisition*. On Day 2, Experiment 1 rats received bilateral CeA infusions of either clonidine or saline (see Drug Infusions) or no infusion (Experiment 2 rats). They were then placed in the experimental chambers and exposed to context-shock pairings. Each animal received two shocks: the first shock was delivered three min after placement into the chamber, and the second occurred 1 min later (four min after placement in the chamber). Rats remained in the chamber for an additional minute after receiving the second shock, yielding a session of five minutes in total.

*Test*. On Day 3, Experiment 2 rats received bilateral CeA infusions of either clonidine or saline (see Drug Infusions) or no infusion (Experiment 1 rats). Animals were then returned to the experimental chambers and tested in the context for 10 minutes in the absence of foot shock, and freezing in the first 2 minutes was analysed.

#### Data Collection and Analysis

Freezing was used to assess conditioned fear. It was defined as the absence of all movement except that required for breathing^37^. Each rat was observed every other second (2 s) and scored for any freezing behaviour (‘*freezing’* or ‘*not freezing*’) by an observer who was naïve to the experimental conditions and group allocations. A percentage score was calculated for the proportion of the total observations scored as *freezing* for each animal. The data was organised using Microsoft Excel (Microsoft Office, 2011) and analysed using a mixed model Analysis of Variance with a between-subject factor of treatment condition (Drug vs. Saline) and a within-subject factor of session time (blocks of 2 min). To maintain the chance of a Type 1 error at alpha = 0.05, the criterion for rejection of the null hypothesis (F_Critical_) was set at F_1,10_ = 4.96.

#### Histology

At the conclusion of testing, all rats were sacrificed via a lethal dose of sodium pentobarbital (i.p.). Rats were then decapitated and their brains were extracted and frozen. Brains were sectioned coronally at 40 μm through the CeA. Every second section was collected on a glass slide and stained with cresyl violet. The location of cannula tips were determined and verified by trained observers under a microscope using the boundaries defined by Paxinos and Watson (2007). Supplementary Figure 1 shows the location of the guide cannula tips for rats infused with clonidine or vehicle. The plotted points represent the ventral point of the cannula track. In experiment 1, four rats were excluded from the statistical analysis: three were excluded due to misplaced cannulas, and one rat died during the surgical procedure. The final group sizes were clonidine, *n* = 6, and saline, *n* = 6. In experiment 2, one rat was excluded due to a misplaced cannula. The final group sizes were clonidine, *n* = 7, and saline, *n* = 8.

### Data availability

The datasets generated during and/or analysed during the current study are available from the corresponding author on reasonable request.

## Electronic supplementary material

Supplementary Information
