# Supplementary material for: α2-adrenoceptor-mediated inhibition in the central amygdala blocks fear-conditioning
Source: Sci Rep. 2017 Sep 15;7:11712. doi: 10.1038/s41598-017-12115-x (PMC5601913; doi:10.1038/s41598-017-12115-x)
Supplement: Supplementary file 1 — Supplementary Information [file 41598_2017_12115_MOESM1_ESM.pdf]

α_2-adrenoceptor-mediated inhibition in the central amygdala blocks fear-conditioning.

N.M. Holmes[2], J.W. Crane[1], M. Tang[2], J. Fam[2], R.F. Westbrook[2] and A.J. Delaney[1*]

Author Affiliation

1. School of Biomedical Sciences, Charles Sturt University, Orange NSW, Australia 2800
2. School of Psychology, University of New South Wales, Sydney NSW, Australia 2052.

*Corresponding Author
Andrew J. Delaney
Senior Lecturer in Anatomy and Physiology
School of Biomedical Sciences,
Charles Sturt University,
Leeds Parade, Orange, NSW,
Australia, 2800
Email – adelaney@csu.edu.au

Supplementary Information

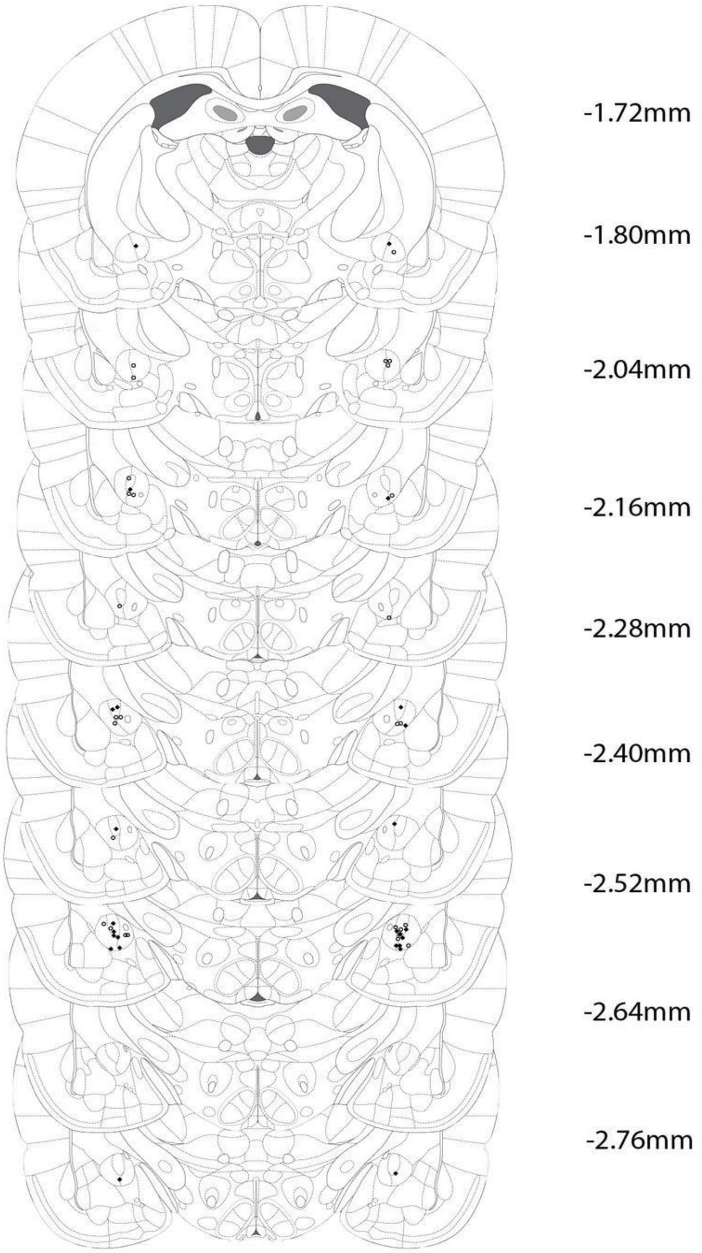

Supplementary Figure 1: Location of cannula placements for clonidine and vehicle infusions into the CeA for context conditioning experiment 1. Only subjects with bilateral cannula placements in the CeA are represented.

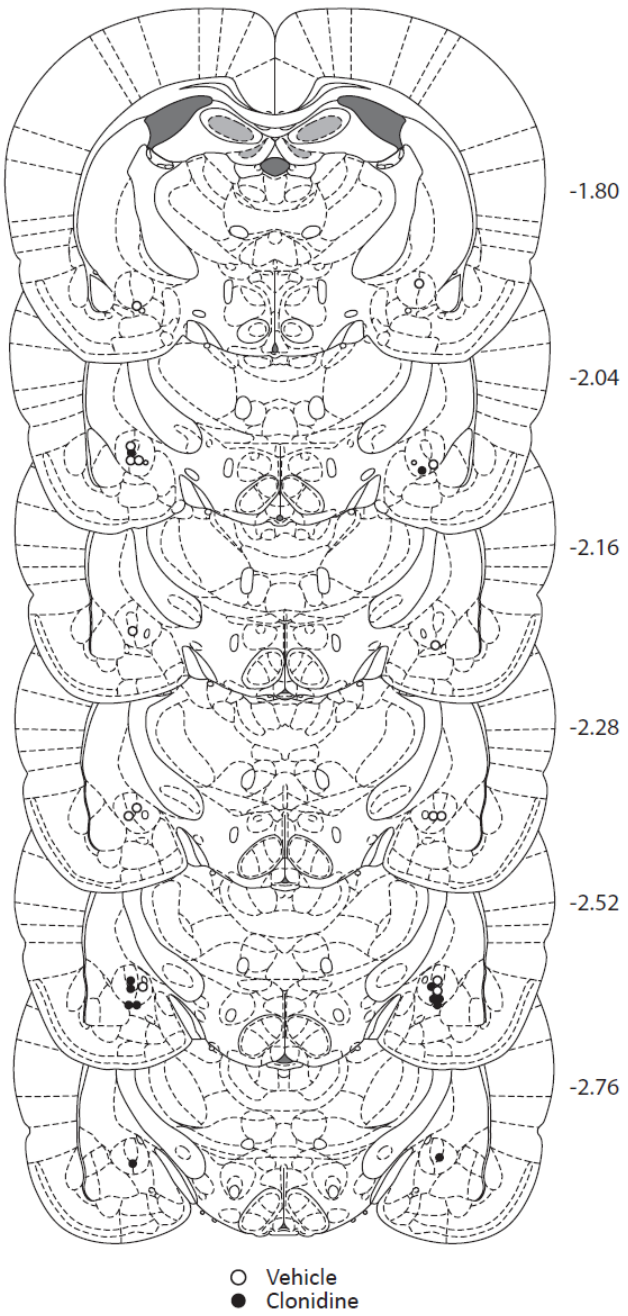

Supplementary Figure 2: Location of cannula placements for clonidine and vehicle infusions into the CeA for context conditioning experiment 2. Only subjects with bilateral cannula placements in the CeA are represented.
